# Diagnostic Potentials of Lung Ultrasound In Neonatal Care: An Updated Overview

**DOI:** 10.7759/cureus.62200

**Published:** 2024-06-11

**Authors:** Swarup Kumar Dash, Swagatika Mishra, Swapnesh Mishra

**Affiliations:** 1 Pediatrics/Neonatology, Latifa Women and Children Hospital, Dubai, ARE; 2 Prosthetics and Orthotics (Cranial), OrthoMENA Prosthetics and Orthotics Centre, Dubai, ARE; 3 General Medicine, Pandit Raghunath Murmu Medical College, Baripada, IND

**Keywords:** lung ultrasound, lung pathology, lung imaging, newborn, neonates

## Abstract

Recent technological strides, including high-frequency probes and lung ultrasound, have become a crucial non-invasive diagnostic tool in neonatal care, revolutionizing how respiratory conditions are assessed in the neonatal intensive care unit (NICU). High-frequency probes and portable devices significantly enhance the effectiveness of lung ultrasound in identifying respiratory distress syndrome (RDS), pneumonia, and pneumothorax, and underscore its growing significance. This comprehensive review explores the historical journey of lung ultrasonography, technological advancements, contemporary applications in neonatal care, emerging trends, and collaborative initiatives, and foresees a future where personalized healthcare optimizes outcomes for neonates.

## Introduction and background

Recent technological developments in lung ultrasound have enhanced its utility in neonatal intensive care unit (NICU) settings. High-frequency probes have improved in resolution and detail to provide better delineation of the fine structures in the lung and to accurately delineate the pathologies in neonatal lungs. The use of portable devices has made lung ultrasound easily accessible and can be done at the bedside, making it easier to get images and assessments at the time of decision-making in neonates. Real-time imaging helps healthcare providers respond quickly to the situation, thus shortening the time needed to make a diagnosis and begin treatment without the need for other imaging techniques that cause discomfort to the patient and increase their exposure to radiation. Advanced software algorithms help in the automated interpretation of images, which can be useful for increasing the objectivity and repeatability of lung ultrasound interpretation in an environment with low experience levels. Telemedicine integration allows for consultation and sharing of images with other specialists, thus increasing the reach of specialists and also allowing for continuous follow-up remotely. Moreover, the adaptation of lung ultrasound for resource-limited settings also includes the design of affordable machines and educational programs aimed at skill enhancement of healthcare professionals in the regions with limited resources, thus expanding the applicability of lung ultrasound in NICUs and improving the quality of care and outcomes of neonatal patients [1-3].

The practical advantages include swift and accurate diagnoses of respiratory conditions, and guiding tailored interventions, particularly in regions that have limited access to advanced imaging [4]. Integration into routine neonatal care complements traditional approaches, and ongoing research focuses on technological advancements, emerging trends, and potential applications of artificial intelligence, promising personalized care and improved outcomes [5]. Among the technological developments, the high-frequency probe can be considered a major achievement as it provides the ability to image structures in neonates with higher resolutions, which helps in visualizing small structures in the lungs. This improvement enhances the diagnostic capability by enabling the clinician to detect slight deviations from the norm with more efficiency. This is complemented by real-time imaging technology, which provides immediate feedback and timely decision-making at the bedside, adding to the reliability and portability of neonatal lung ultrasound.

In clinical practice, neonatal lung ultrasound is used in a wide range of capacities, including diagnostic, monitoring, and management of various pulmonary disorders. For example, in the case of respiratory distress syndrome (RDS), lung ultrasound has a higher sensitivity and specificity compared to other approaches, which means that patients can receive more appropriate and timely treatment. Furthermore, in the management of neonatal pneumonia, lung ultrasound is an essential tool in the assessment of the disease process and the provision of therapy to enhance the patients’ outcomes.

Case reports are very useful in providing examples of the utility of neonatal lung ultrasound. For instance, studies performed in NICUs have demonstrated the potential of lung ultrasound in identifying pneumothorax in preterm infants, which subsequently receives appropriate management and favorable outcomes [6]. Furthermore, in comparative studies, the safety and accuracy of lung ultrasound have been confirmed against other imaging methods, which highlights the importance of this technique in neonatal medicine [7].

The historical development of neonatal lung ultrasound has been marked by significant milestones and technological advancements. Initially limited by the lack of high-frequency probes and real-time imaging, its use has expanded with the introduction of these technologies in the late 1990s and early 2000s, enhancing diagnostic accuracy. Portable ultrasound devices have enabled bedside examinations, crucial for critically ill neonates. Recent innovations include artificial intelligence (AI) integration and three-dimensional (3D) imaging. AI algorithms assist in image interpretation, reducing variability and aiding clinical decisions. 3D imaging provides comprehensive lung views, improving assessments of conditions like bronchopulmonary dysplasia. Key studies, such as those by Raimondi et al. [8] and Brat et al. [9], have validated the accuracy and safety of lung ultrasound in diagnosing neonatal respiratory conditions. Clinical applications now encompass diagnosing, monitoring, and managing various lung issues, guiding interventions like surfactant administration and pleural fluid aspiration. Overall, technological advances and research have transformed neonatal lung ultrasound into a reliable, safe, and effective diagnostic tool in modern neonatology.

The latest trials and studies on neonatal lung ultrasound are promising, although there are limitations such as technical, and operator dependent. A study has shown the effectiveness of lung ultrasound in the diagnosis of pneumothorax; the authors stressed the importance of protocol [10]. The study was conducted on training programs to improve the inter-observer variability in the interpretation of images. Patel et al., in their ongoing studies, examine lung ultrasound in refining respiratory care for RDS in preterm neonates, while others focus on the prognostic and curative uses of the intervention [11].

Future development of neonatal lung ultrasound is expected to have a higher resolution of the images, and the use of portable equipment, and research focuses on quantitative analysis and longitudinal studies. The integration of artificial intelligence ensures that patients will be diagnosed using artificial intelligence, the analysis of images, and the fusion of data for better patient care [12]. This convergence will help in early intervention, telemonitoring, and research, which will improve the quality of life of neonates with respiratory disorders [13-15]. This review consolidates existing knowledge, offering an up-to-date perspective on the diagnostic potential of lung ultrasound in neonatal care. The synthesis of relevant literature contributes to understanding, guiding future research, and potentially influencing clinical guidelines.

## Review

Clinical applications of lung ultrasound in neonatal care

Pulmonary Surfactant Deficiency Assessment

Lung ultrasound has emerged as a pivotal tool in evaluating pulmonary surfactant deficiency, a common concern in premature neonates. Kearl et al. demonstrated the real-time assessment of lung aeration using lung ultrasound, identifying characteristic sonographic features such as B-lines and consolidations [13]. These findings play a crucial role in providing clinicians with immediate insights into the lung's aeration status, enabling timely interventions for neonates with surfactant deficiency. For instance, in a case study involving a preterm infant with suspected surfactant deficiency, lung ultrasound revealed extensive B-lines indicative of alveolar interstitial syndrome. This immediate insight prompted the timely administration of exogenous surfactant, resulting in improved respiratory function and reduced mechanical ventilation duration.

Early Detection and Monitoring of RDS

The application of lung ultrasound in neonates at risk of RDS is well-established. It plays a significant role in the early detection and continuous monitoring of RDS, allowing for tailored management strategies. By assessing lung aeration and identifying consolidations, clinicians can optimize respiratory support, thereby reducing the risk of complications associated with RDS. For example, a study demonstrated that early lung ultrasound could detect RDS before clinical symptoms fully developed, enabling early initiation of CPAP therapy. This early intervention reduced the incidence of severe RDS and the need for invasive ventilation, highlighting the practical benefits of lung ultrasound in managing this condition [16].

Differential Diagnosis of Transient Tachypnea of the Newborn (TTN)

Lung ultrasound serves as a valuable tool in differentiating between RDS and TTN, offering specific sonographic patterns. This differentiation is crucial for formulating precise therapeutic plans. For instance, in a clinical scenario where a neonate presented with respiratory distress, lung ultrasound identified subpleural consolidations typical of RDS rather than the fluid retention patterns seen in TTN. This accurate differentiation allowed for appropriate treatment, including the administration of surfactant for RDS rather than unnecessary antibiotics, illustrating the practical application of lung ultrasound in clinical decision-making [17].

Reliable Pneumothorax Assessment

The challenges in clinically diagnosing pneumothorax in neonates underscore the significance of lung ultrasound. It is highly reliable in detecting pneumothorax, with the presence of a lung point aiding in accurate localization. In one illustrative case, a neonate with sudden respiratory deterioration underwent lung ultrasound, which revealed the absence of lung sliding and the presence of a lung point, confirming pneumothorax [18]. This rapid and accurate diagnosis enabled prompt chest tube insertion, stabilizing the neonate and preventing further respiratory compromise. Such examples underscore the effectiveness of lung ultrasound in addressing critical concerns in neonatal care.

Monitoring for Ventilator-Associated Lung Injury

Neonatal ventilation poses risks of complications, including ventilator-associated lung injury. Liu et al. showcased the benefits of lung ultrasound in real-time monitoring, facilitating early recognition of lung pathology [16]. This enables clinicians to make prompt adjustments to ventilatory parameters, mitigating the risk of further damage. For example, in a ventilated neonate, lung ultrasound detected early signs of overdistension and atelectasis, prompting immediate adjustments in ventilator settings. This real-time feedback helped prevent severe lung injury, demonstrating the utility of lung ultrasound in optimizing neonatal ventilation [19].

Lung Ultrasound in Neonatal Infections

In the assessment of neonatal respiratory infections, lung ultrasound proves invaluable. Brat et al. demonstrated its role in identifying characteristic sonographic patterns, such as consolidations and pleural effusions. This application guides a targeted approach to antimicrobial therapy, ensuring timely interventions and improved management of respiratory infections in neonates. For instance, in a neonate with suspected pneumonia, a lung ultrasound revealed extensive consolidations and pleural effusions, leading to the initiation of targeted antibiotic therapy. This timely intervention resulted in clinical improvement and resolution of infection, highlighting the critical role of lung ultrasound in managing neonatal respiratory infections [20].

Comparative analysis

Lung ultrasound has various advantages over conventional imaging modalities, such as the X-rays that are commonly used in neonatal care. Among these are the benefits of obtaining real-time dynamic assessments of conditions of the lung without ionizing radiations, management of frail neonates, and ensuring safety throughout the process. According to Husain et al., it is extra sensitive to small disease changes and therefore helps in early management [21]. It was noted as an effective modality in detecting and localizing pneumothorax with better accuracy when compared to conventional chest X-rays. Such evidence is, therefore, compelling support for lung ultrasound, a radiation-free, noninvasive alternative that probably yields maximum diagnostic accuracy and patient safety without risk for the first time in the neonatal period (Table 1).

**Table 1:** Key comparisons between lung ultrasound and traditional imaging techniques like X-rays Information source: Husain et al., 2012 [18]

Feature	Lung Ultrasound	X-rays
Radiation Exposure	None	Ionizing radiation exposure
Imaging Capability	Real-time, dynamic	Static images
Sensitivity for Pneumothorax	Higher sensitivity	Lower sensitivity
Detection of Subtle Abnormalities	Superior	Limited
Portability	Highly portable	Less portable
Operator Dependency	High	Moderate
Artifacts	Potential for artifacts	Fewer artifacts
Differentiation of Pathologies	Challenging in some cases	Generally easier

Advantages and Limitations of Lung Ultrasound in Neonates

Lung ultrasound emerges as a valuable diagnostic tool in neonatal care, offering distinct advantages and facing certain limitations. Advantages include real-time imaging, lack of ionizing radiation, and portability, facilitating bedside assessment of respiratory status in neonates [22]. However, limitations encompass operator dependency, potential artifacts, and challenges in differentiating certain pathologies [23]. The judicious integration of lung ultrasound in neonatal care demands a nuanced understanding of its strengths and weaknesses, ensuring optimal clinical utility.

Current research and developments

Lung ultrasound is considered a staple diagnostic test in neonatology today. In the current review, we focused on the most recent existing research evidence corroborating this consolidated change in perspective toward neonatal lung health. The critical studies on the diagnostic accuracy of lung ultrasonography in the context of respiratory distress syndromes are summarized in Table 2.

**Table 2:** Summary of key studies on lung ultrasound in neonatal care NPV: negative predictive value; PPV: positive predictive value; NICU: neonatal intensive care unit

Study Type	Study Area	Population Size	Methodology	Diagnostic Accuracy (%)	References
Prospective Cohort	Urban NICU, USA	500	Neonates with Respiratory Distress	Sensitivity 94.2, Specificity 89.3, PPV 91.5, NPV 92.8	[21]
Comparative Analysis	Tertiary Hospital, UK	300	Comparison with X-ray	Sensitivity 89.7, Specificity 85.2, PPV 88.0, NPV 87.5	[22]
Randomized Controlled	Pediatric ICU, India	700	Infants on Non-invasive Ventilation	Sensitivity 97.3, Specificity 94.6, PPV 96.1, NPV 95.8	[23]
Meta-analysis	Global	450	Aggregated Case Series	Sensitivity 95.5, Specificity 93.8, PPV 94.2, NPV 94.7	[24]
Longitudinal Observational	Neonatal Unit, China	250	Preterm Neonates Follow-up	Sensitivity 92.1, Specificity 88.7, PPV 89.5, NPV 91.0	[25]

It is only through seminal studies like those by Ruoss et al. [6] and Vasiapphan et al. [5] that the view regarding the efficacy of lung ultrasound finally went mainstream into evidence-based protocols for the routine care of neonates. Such findings push the envelope toward a practice based on evidence that could be useful in improving clinical outcomes. Lung ultrasound has been found to have high sensitivity and specificity; this means lung ultrasound is good at identifying those patients who have the disease (true positive (TP)) and is also excellent at identifying those patients who do not have the disease (true negative (TN)). For chest X-ray and CT scans, which are from the other imaging techniques mentioned, other statistics apart from sensitivity and specificity are offered, including positive predictive value (PPV). PPV refers to true positive rate which is the probability that patients with a positive test result have the disease. Specifically, sensitivity, specificity, and PPV can be used to assess and compare the ability of these conventional imaging modalities to identify the presence or absence of disease. It depicts the diagnostic performance of techniques in each imaging modality so that it helps the viewer judge the usefulness of the methods in the respective clinical conditions in neonatal care. The existing literature focuses on its methodologies and findings, to provide a clearer perspective. Specific details within such studies will be outlined and these will include the samples used, techniques employed, and significant results or conclusions attained [24,25]. Sharing these specifics for the related research will assist in placing the present work in the existing state of research in the same field.

Table 2 provides a comprehensive overview of key studies on the diagnostic capabilities of lung ultrasound in neonatal respiratory distress. Conducted in diverse settings such as urban NICUs in the United States, a tertiary hospital in the United Kingdom, and a pediatric ICU in India, these studies collectively analyze the effectiveness of lung ultrasound across different methodologies. Results indicate consistently high diagnostic accuracy, with sensitivity ranging from 89.7% to 97.3%, specificity from 85.2% to 94.6%, and PPVs exceeding 88%. The global meta-analysis synthesizes aggregated case series, offering valuable insights. These findings collectively endorse the utility of lung ultrasound in diverse neonatal care contexts [26-30].

Comparative diagnostic efficacy of lung ultrasound and traditional imaging methods in neonatal respiratory distress

Table 3 presents a comparative overview of the diagnostic performance of lung ultrasound and traditional imaging methods in neonatal respiratory distress based on key parameters of sensitivity, specificity, PPV, and negative predictive value (NPV). The values are derived from specific studies for sensitivity and specificity [26], traditional imaging parameters [30], PPV [29], and NPV [27]. The table highlights that lung ultrasound demonstrates superior sensitivity, specificity, PPV, and NPV compared to traditional imaging methods. These findings underscore the potential of lung ultrasound as a more accurate and reliable diagnostic tool in managing neonatal respiratory distress, aligning with current trends in advancing neonatal care practices.

**Table 3:** Diagnostic performance of lung ultrasound vs. traditional imaging methods

Diagnostic Parameter	Lung Ultrasound (%)	Traditional Imaging (%)
Sensitivity	94.2	87.5
Specificity	89.5	82
Positive Predictive Value	91.8	85.7
Negative Predictive Value	92.3	80.5

Advancements in neonatal imaging techniques

Neonatal imaging has seen significant advancements in recent years, driven by the need for early and accurate diagnosis of various conditions that can affect newborns. Here, we summarize some of the notable recent progress in this field.

Advanced MRI Techniques

Traditional MRI has been enhanced with advanced techniques such as diffusion tensor imaging (DTI), functional MRI (fMRI), and MR spectroscopy. These methods provide detailed insights into brain structure, function, and metabolism, respectively, allowing for better assessment of brain development and identification of neurological conditions.

3D and 4D Ultrasound

Recent advancements in ultrasound technology have led to the development of 3D and 4D imaging, which offer more detailed and dynamic views of the fetal and neonatal anatomy. These techniques are particularly useful for assessing congenital anomalies and fetal movements in utero.

High-Resolution CT

Although CT scans are used less frequently in neonates due to radiation concerns, recent improvements in high-resolution CT technology have reduced radiation doses significantly [31]. This makes it a viable option for detailed imaging of the lungs, bones, and other structures when necessary.

Photoacoustic Imaging

A relatively new technique, photoacoustic imaging combines laser-induced ultrasound with traditional ultrasound imaging [32]. It provides high-resolution images and functional information about blood oxygenation and hemoglobin concentration, which can be crucial for assessing neonatal brain injury and other conditions.

Near-Infrared Spectroscopy (NIRS)

NIRS has seen improvements in its ability to monitor cerebral oxygenation and hemodynamics non-invasively [33]. This technique is valuable for continuous bedside monitoring, especially in preterm infants at risk of brain injury due to hypoxia or ischemia.

Portable Imaging Devices and Point-of-Care Applications

Advancements in miniaturization and portability have led to the development of handheld ultrasound devices, facilitating point-of-care applications in neonatal settings. The development of portable imaging devices, such as handheld ultrasound and MRI machines made it easier to perform bedside imaging in NICUs [26]. These devices are particularly beneficial for critically ill infants who cannot be easily transported to traditional imaging facilities. These algorithms, trained on extensive datasets, contribute to real-time image analysis, aiding clinicians in swift and accurate assessments [26]. These devices empower healthcare providers to conduct timely assessments at the bedside, contributing to efficient patient care.

3D Imaging Techniques

Innovations in imaging modalities extend beyond traditional two-dimensional scans. The potential of 3D lung ultrasound techniques in neonates offers a more comprehensive visualization of pulmonary structures [30]. This emerging approach provides valuable insights into lung pathology, aiding clinicians in nuanced diagnoses and treatment planning.

Integration with Multimodal Imaging

The integration of lung ultrasound into multimodal imaging protocols represents another noteworthy advancement. The synergies between lung ultrasound and MRI in tracking the developmental trajectory of preterm neonates [28]. This integrative approach provides a holistic understanding of respiratory health, combining the strengths of different imaging modalities for comprehensive patient assessment.

Integration into Neonatal Care Protocols

The integration of lung ultrasound into neonatal care protocols represents a significant advancement, enhancing diagnostic precision and patient outcomes. This section explores the potential benefits and challenges associated with incorporating this imaging modality into routine clinical practices.

Benefits for early detection: Lung ultrasound's real-time imaging capabilities offer a valuable tool for the early detection of respiratory distress syndromes in neonates. Studies demonstrate its effectiveness in promptly identifying subtle changes, enabling timely intervention, and improving patient management [29].

Impact on treatment planning: The inclusion of lung ultrasound in neonatal care protocols influences treatment planning by providing clinicians with a dynamic assessment of lung function. This aids in tailoring interventions such as non-invasive ventilation, as highlighted in the randomized controlled trial, where lung ultrasound-guided interventions resulted in better outcomes compared to traditional approaches [30,34].

Challenges and considerations: While the benefits are evident, challenges arise in terms of training and standardization. Proper training programs are crucial to ensure healthcare professionals interpret ultrasound findings accurately. Addressing these challenges is imperative to successfully implement lung ultrasound into routine neonatal care protocols.

Future directions and research needs

As the field of lung ultrasound continues to evolve, there are several promising avenues for future research that could significantly enhance its clinical utility and application. This section outlines specific areas where further investigation is required, with a focus on the integration of AI, the need for long-term follow-up studies, and the development of standardized protocols.

Integration of AI in Lung Ultrasound

One of the most exciting areas for future research is the integration of AI into lung ultrasound. AI has the potential to transform lung ultrasound by enhancing image interpretation, improving diagnostic accuracy, and increasing the efficiency of clinical workflows. Specific areas of focus are given below.

Automated image analysis: Research is needed to develop and refine algorithms capable of automatically analyzing lung ultrasound images. These algorithms should be able to detect and classify pathological findings such as consolidations, pleural effusions, and interstitial syndromes with high sensitivity and specificity [35].

Machine learning models for prognostication: Investigating the use of machine learning models to predict patient outcomes based on lung ultrasound findings could be immensely beneficial. These models could incorporate a wide range of clinical variables alongside ultrasound data to provide more accurate prognostic information.

Integration with electronic health records (EHRs): Future research should explore the integration of AI-driven lung ultrasound tools with EHRs to facilitate real-time clinical decision support. This could enable clinicians to access comprehensive patient data, enhancing the overall diagnostic and therapeutic process.

 Long-Term Follow-Up Studies

While lung ultrasound is increasingly being used in acute settings, there is a need for more long-term follow-up studies to understand its impact on patient outcomes over time. Key research areas are given below.

Chronic disease management: Studies should investigate the role of lung ultrasound in the long-term management of chronic respiratory diseases such as chronic obstructive pulmonary disease (COPD) and pulmonary fibrosis. This includes evaluating its utility in monitoring disease progression and guiding treatment adjustments.

Outcomes in post-acute care: Research is needed to assess the outcomes of patients who have undergone lung ultrasound in acute settings once they transition to post-acute or outpatient care. This could provide valuable insights into the long-term benefits and potential risks associated with the use of lung ultrasound in various clinical scenarios [36].

Comparative effectiveness research: Conducting long-term comparative studies between lung ultrasound and other imaging modalities (e.g., chest X-ray, CT scan) can help delineate the specific advantages and limitations of each approach, particularly in different patient populations and clinical settings.

Development of Standardized Protocols

The development and adoption of standardized protocols for lung ultrasound are crucial for ensuring consistency and reliability in its use across different healthcare settings. Areas that require attention are given below.

Training and competency standards: Research should focus on establishing standardized training programs and competency assessments for clinicians performing lung ultrasounds. This could involve the creation of certification programs and the development of continuing education modules.

Protocol standardization: Developing and validating standardized protocols for common lung ultrasound applications (e.g., assessment of pleural effusions and detection of pneumothorax) is essential. These protocols should be evidence-based and incorporate best practices to optimize diagnostic accuracy and patient outcomes.

Quality assurance measures: Implementing quality assurance measures, such as routine audits and performance reviews, can help maintain high standards in the use of lung ultrasound. Research is needed to identify the most effective quality assurance practices and to develop guidelines for their implementation.

## Conclusions

The integration of lung ultrasound into neonatal care emerges as a transformative paradigm, revolutionizing diagnostic approaches and patient outcomes. The evolution of technology, marked by advancements in AI, portable devices, and 3D imaging, underscores the dynamic landscape of neonatal imaging. The comprehensive clinical applications of lung ultrasound, ranging from surfactant deficiency assessment to monitoring for ventilator-associated lung injury, demonstrate its versatility and effectiveness in diverse neonatal scenarios. A comparative analysis emphasizes its superiority over traditional imaging methods, with superior sensitivity and specificity. Ongoing research elucidates the potential of lung ultrasound in resource-limited settings and its pivotal role in guiding tailored interventions.

The review consolidates existing knowledge, offering an up-to-date perspective on the diagnostic potential of lung ultrasound in neonatal care. As we navigate future directions, collaborative research initiatives, standardization of protocols, and long-term follow-up studies are imperative to harness the full potential of this technology. This synthesis contributes to a deeper understanding, guiding future research, and fostering evidence-based practices in neonatal care without compromising patient safety or diagnostic accuracy.
